# The Adaptive Renal Response for Volume Homeostasis During 2 Weeks of Dapagliflozin Treatment in People With Type 2 Diabetes and Preserved Renal Function on a Sodium-Controlled Diet

**DOI:** 10.1016/j.ekir.2022.02.023

**Published:** 2022-03-04

**Authors:** Rosalie A. Scholtes, Marcel H.A. Muskiet, Michiel J.B. van Baar, Anne C. Hesp, Peter J. Greasley, Ann Hammarstedt, Cecilia Karlsson, Karen M. Hallow, A.H. Jan Danser, Hiddo J.L. Heerspink, Daniël H. van Raalte

**Affiliations:** 1 Diabetes Center, Department of Internal Medicine, Amsterdam University Medical Centers, location VU University Medical Center, Amsterdam, The Netherlands; 2 Research and Early Development, Cardiovascular, Renal and Metabolism (CVRM), BioPharmaceuticals R&D, AstraZeneca, Gothenburg, Sweden; 3 Late-Stage Development, Cardiovascular, Renal and Metabolism (CVRM), BioPharmaceuticals R&D, AstraZeneca, Gothenburg, Sweden; 4 School of Chemical, Materials, and Biomedical Engineering, University of Georgia, Athens, Georgia; 5 Division of Pharmacology and Vascular Medicine, Department of Internal Medicine, Erasmus MC, Rotterdam, The Netherlands; 6 Department of Clinical Pharmacy and Pharmacology, University of Groningen, University Medical Center Groningen, Groningen, The Netherlands

**Keywords:** body fluid homeostasis, renal adaptive mechanisms, SGLT2 inhibitors, type 2 diabetes, water conservation

## Abstract

**Introduction:**

Proximal tubule sodium uptake is diminished following sodium glucose cotransporter 2 (SGLT2) inhibition. We previously showed that during SGLT2 inhibition, the kidneys adapt by increasing sodium uptake at distal tubular segments, thereby maintaining body sodium balance. Despite continuous glycosuria, we detected no increased urine volumes. We therefore assessed the adaptive renal responses to prevent excessive fluid loss.

**Methods:**

We conducted a mechanistic open-label study in people with type 2 diabetes mellitus with preserved kidney function, who received a standardized sodium intake (150 mmol/d) to evaluate the effects of dapagliflozin on renin-angiotensin-aldosterone system (RAAS) hormones, volume-related biomarkers, urinary albumin-to-creatinine ratio (UACR), and estimated glomerular filtration rate (eGFR), at start of treatment (day 4), end of treatment (day 14), and follow-up (day 18).

**Results:**

A total of 14 people were enrolled. Plasma renin and angiotensin II and urinary aldosterone and angiotensinogen were acutely and persistently increased during treatment with dapagliflozin. Plasma copeptin level was numerically increased after 4 days (21%). Similarly, fractional urea excretion was significantly decreased at start of treatment (−17%). Free water clearance was significantly decreased after 4 days (−74%) and 14 days (−41%). All changes reversed after dapagliflozin discontinuation.

**Conclusion:**

Dapagliflozin-induced osmotic diuresis triggers kidney adaptive mechanisms to maintain volume and sodium balance in people with type 2 diabetes and preserved kidney function. ClinicalTrials.gov (identification: NCT03152084).

## Graphical abstract

**Figure:**
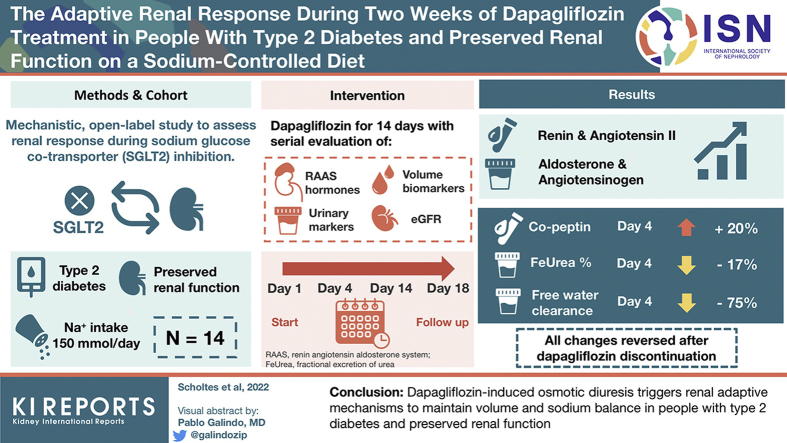

SGLT2 inhibitors, such as dapagliflozin, lower blood glucose levels by inhibiting glucose reuptake in the proximal tubule, resulting in urinary glucose excretion.^1^ SGLT2 inhibitors improve renal outcomes in people with chronic kidney disease independent of the presence of diabetes.^2^, ^3^, ^4^, ^5^, ^6^, ^7^ The most likely explanation of this renal benefit is that SGLT2 inhibitors reduce intraglomerular pressure due to activation of tubuloglomerular feedback secondary to increased sodium and chloride delivery to the macula densa.^8^^,^^9^ Owing to reduced proximal tubule sodium uptake, the kidneys adapt by increasing sodium uptake at more distal tubular segments, thereby maintaining body sodium balance. As such, we recently showed that in people with type 2 diabetes and preserved kidney function on a strictly controlled sodium diet, dapagliflozin did not alter 24-hour sodium excretion.^10^ Interestingly, despite ongoing glycosuria, dapagliflozin did not cause significant changes in 24-hour urinary volumes,^10^ reflecting adaptive kidney mechanisms to maintain body fluid volumes. So far, it is not clear which renal-specific compensatory mechanisms are activated, but RAAS activation, increments in copeptin secretion, as well as retention of urea may be among potential mechanisms that mediate this response.^11^

The aim of this study was to assess these integrated kidney responses to dapagliflozin therapy which prevent excessive loss of water and sodium. Second, we measured the immediate effects of dapagliflozin on eGFR and urinary albumin excretion. Data were collected during a strictly sodium-controlled diet. Blood and 24-hour urine sampling were carried out at baseline, following acute and more chronic therapy and following a washout period.

## Methods

### Trial Design

This was a prespecified exploratory analysis of the DAPASALT trial, a phase 4, multicenter, open-label, mechanistic interventional study conducted between July 2017 and March 2020, primarily designed to assess the natriuretic effect of 2-week treatment with dapagliflozin, which was published recently.^10^ The study was originally designed with 3 strata, consisting of people with type 2 diabetes and impaired kidney function (stratum 1), people with type 2 diabetes and preserved kidney function (stratum 2), and people without diabetes with impaired kidney function (stratum 3) with a total sample size of 51 people; 17 people per stratum. Here, we present data on the completed stratum of people with type 2 diabetes and preserved kidney function. People were recruited at the Amsterdam University Medical Centers, location VUMC, Amsterdam, and at Ziekenhuisgroep Twente, Almelo, the Netherlands. The study protocol, protocol amendments, and all other protocol-specific documents were reviewed and approved by local authorities and the medical ethical review board of the participating centers. The study complied with the Declaration of Helsinki and Good Clinical Practice guidelines and was registered at ClinicalTrials.gov (identification: NCT03152084).

### Study Population

We included Caucasian, Asian, or Middle-Eastern men and women using contraception or who were surgically sterile or postmenopausal, aged 18 to ≤80 years, who were diagnosed with having type 2 diabetes and had a glycated hemoglobin ranging from 6.5% (48 mmol/mol) to <10% (<86 mmol/mol), and were treated with a stable dose of metformin, or sulfonylurea, or a combination of metformin and sulfonylurea for at least 3 months before enrollment. People had to have a preserved kidney function defined as an eGFR between >90 and ≤130 ml/min per 1.73 m^2^ for people aged 59 years or younger, between >85 and ≤130 ml/min per 1.73 m^2^ for people aged 60 to 69 years, and between >75 and ≤130 ml/min per 1.73 m^2^ for people aged ≥70 years. Furthermore, a stable dose of an angiotensin receptor blocker for at least 6 weeks was mandatory to create a homogeneous cohort using a similar class of drugs to inhibit the RAAS. People should have a stable 24-hour urinary sodium excretion on 2 successive days (<20% difference between days −3 and −2) before inclusion. People with a history of unstable or rapidly progressing kidney disease, albumin-to-creatinine ratio >1000 mg/g, symptoms of urinary retention, use of a pacemaker or other implanted electronic devices, type 1 diabetes, systolic blood pressure (BP) ≥180 mm Hg or diastolic BP ≥110 mm Hg, or cardiovascular/vascular disease within 3 months before screening, were excluded. During the study use of any other glucose-lowering drugs besides metformin and sulfonylurea, use of angiotensin-converting enzyme inhibitors, use of nonsteroidal anti-inflammatory drugs, or use of diuretics within 2 weeks before the study was not allowed. People were recruited from study databases and by advertisements in local newspapers. Written informed consent was obtained from all people before any trial-related activities.

### Intervention

Eligible people received dapagliflozin 10 mg tablets once daily for 14 ± 1 days. People were instructed to take their study medication in the morning.

### Outcome Measures

The primary objective of the study was to assess the natriuretic effect of dapagliflozin as published previously.^10^^,^^12^ Secondary objectives included the effect of dapagliflozin on changes in 24-hour UACR from baseline to day 4 and to end of treatment (days 12–14). Changes in RAAS biomarkers (plasma renin and angiotensin II, urinary renin, aldosterone, and angiotensinogen) and volume-related biomarkers (plasma copeptin, plasma urea and fractional urea excretion, and urine osmolality) were exploratory end points measured at baseline, start of treatment, end of treatment, and during follow-up and are reported here. Change from end of treatment to follow-up in 24-hour UACR was considered a *post hoc* exploratory end point.

### Procedures and Follow-Up Visits

#### Run-In Period (Day −6 to Day −1)

People received food boxes from Sodexo (Rotterdam, the Netherlands) with a daily sodium content of 150 mmol. The dietary requirements started on day −6, and people were required to follow these instructions throughout the entire study period until and including day 18 (Supplementary Figure S1). Participants were obligated to complete food questionnaires starting from day −6 to record any deviation in intake from the provided food boxes and liquid intake. Besides the required products from the food boxes, people were allowed to consume nonstudy food products that did not contain sodium. These products were also recorded in the food questionnaires. At baseline (day −3 to day −1), 24-hour urine samples were collected. People were given instructions beforehand about how to collect 24-hour urine samples. People in whom 24-hour sodium excretion did not differ by >20% from day −3 to day −2 were considered to be adherent to the diet and could proceed to the active treatment period.

#### Treatment Period (Day 1–Day 14)

At day 1, day 4, day 5, day 13, and day 14, inpatient study visits were scheduled. In total, 4 serial 24-hour urine samples were collected at days 1 to 4 followed by 3 serial 24-hour urine collections at days 12 to 14. Blood samples were obtained in fasting condition in the morning of days 1, 4, and 14.

#### Follow-Up Period (Day 15–Day 19)

At days 15 to 17, 3 serial 24-hour urine samples were collected (follow-up). At day 18, a final inpatient study visit was scheduled and fasted blood samples were obtained.

### Laboratory Measurements

All samples were measured by standard in-house assays at Covance (Geneva, Switzerland), Amsterdam University Medical Center, and Ziekenhuisgroep Twente (hemoglobin, hematocrit, urea, glucose, sodium, glycated hemoglobin, creatinine as well as urinary concentrations of sodium, potassium, glucose, creatinine, albumin, and urea). Plasma copeptin was measured by LS Bio, copeptin enzyme-linked immunosorbent assay in human plasma (Covance Laboratories, Inc., Greenfield, IN). Urine osmolality was calculated from the following urinary molecule concentrations: 2× [sodium + potassium] + urea + glucose. Plasma renin was measured using a radioimmunometric assay (Cisbio, Saclay, France). Urinary renin was measured using an in-house enzyme-kinetic assay that quantifies angiotensin I generation in the presence of excess sheep angiotensinogen.^13^ To convert angiotensin I-generating activity to renin concentration, a conversion factor was used, based on the fact that 1 ng angiotensin I/ml per hour corresponds with 2.6 pg renin/ml.^14^ Urinary angiotensinogen was measured by commercial enzyme-linked immunosorbent assay (IBL International, Hamburg, Germany). Plasma angiotensin II was measured by an in-house radioimmunoassay.^15^

### Statistical Analysis

Sample size calculations were performed for each of the 3 study-strata individually. For full details, refer to the previously published manuscript.^10^ Baseline characteristics were summarized using mean and SD or proportions where appropriate. Longitudinal repeated measures models were used for the change versus the baseline values. The model included time point as a fixed effect, interaction term between time point and baseline, and continuous baseline value as covariates. In case of skewed distributed variables, geometric mean and corresponding percentage change (95% CI) were reported at each time point. Statistical analyses were performed using SAS software version 9.4 (SAS Institute, Cary, NC).

## Results

### Subject Characteristics

A total of 31 people gave written informed consent. A total of 17 people started treatment with dapagliflozin. Due to >20% difference in urinary sodium excretion at day −3 and day −2, and missing urine volume for 24-hour urine collection at day −2, 2 people were excluded from the analysis before database lock. After database lock, 1 additional patient was excluded owing to nonadherence to dapagliflozin. Efficacy analyses were therefore performed in 14 people, and their baseline characteristics are reported in Table 1.

**Table 1:** Baseline characteristics

Characteristics	Study participants (*N* = 14)
Age, yr	63.9 (7.9)
Male sex, *n* (%)	9 (64.3)
Race, *n* (%)	
White	13 (92.9)
Asian	1 (7.1)
Diabetes duration, yr	10.2 (5.2)
Body weight, kg	98.7 (15.9)
BMI, kg/m^2^	31.9 (4.2)
Fasting plasma glucose, mmol/l	8.1 (1.4)
HbA1c, %	7.2 (0.6)
HbA1c, mmol/mol	55 (6.6)
Systolic blood pressure, mm Hg	128.6 (13.6)
Diastolic blood pressure, mm Hg	74.7 (7.5)
eGFR (CKD-EPI), ml/min per 1.73 m^2^	94.3 (10.9)
UACR, mg/mmol	0.8 [0.5–2.8]^a^
Hemoglobin, g/L	137.9 (13.0)
Hematocrit, L/L	0.4 (0.0)
Metformin, *n* (%)	14 (100)
Sulfonylurea derivative, *n* (%)	5 (35.7)

BMI, body mass index; CKD-EPI, Chronic Kidney Disease-Epidemiology Collaboration; eGFR, estimated glomerular filtration rate; HbA1c, glycated hemoglobin; UACR, urinary albumin-to-creatinine ratio.

Data are mean (SD) unless otherwise indicated. Blood pressure recorded in supine position.

a Median [25th–75th percentile].

### Adaptive Kidney Response: Secretion of Copeptin, Urea Retention, and Effect on Osmolality and Free Water Clearance

**Figure 1:**
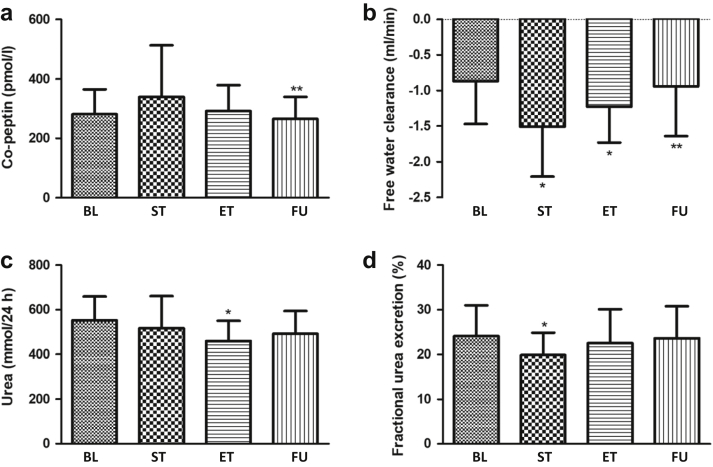
(a) Copeptin, (b) free water clearance, (c) urinary urea excretion, and (d) fractional urea excretion at BL, ST, ET, and FU. ∗Significantly differernt compared with BL. ∗∗Significantly different compared with BU. BL, baseline; ET, end of treatment; FU, follow-up; ST, start of treatment.

Mean baseline plasma copeptin levels numerically increased from baseline (mean: 281.1 pmol/l [SD: 84]), during early dapagliflozin treatment (mean at start of treatment: 339.8 pmol/l [SD: 173], *P* = 0.19; mean at end of treatment: 291.2 pmol/l [SD: 88], *P* = 0.67). During follow-up, plasma copeptin levels significantly decreased compared with end of treatment (mean at day 18: 265.2 pmol/l [SD: 74]; *P* = 0.03; Table 2 and Figure 1).

**Table 2:** Adaptive renal responses (*N* = 14)

	Baseline	Start of treatment^a^			End of treatment^a^			Follow-up^b^		
	Mean (SD) or geometric mean [95% CI]	Mean (SD) or geometric mean [95% CI]	Change (95% CI) or percentage change [95% CI]	*P* value	Mean (SD) or geometric mean [95% CI]	Change (95% CI) or percentage change [95% CI]	*P* value	Mean (SD) or geometric mean [95% CI]	Change (95% CI) or percentage change [95% CI]	*P* value
	Volume-related biomarkers									
Plasma										
Copeptin (pmol/L)	281.1 (84)	339.8 (173)	58.7 (−33 to 150)	0.19	291.2 (88)	11.0 (−44 to 67)	0.67	265.2 (74)	−51 (−97.3 to −4.8)	**0.03**
Osmolality (mOsm/kg)	302 (3)	300 (4)	−1.4 (−3.3 to 0.5)	0.13	301 (5)	−0.9 (−2.7 to 0.9)	0.30	301 (3)	−0.04 (−1.8 to 1.8)	0.96
Urea (mmol/L)	5.4 (1.0)	5.9 (1.4)	0.46 (−0.06 to 0.98)	0.08	5.4 (1.2)	0.0 (−0.5 to 0.6)	0.98	5.0 (1.4)	−0.46 (−1.1 to 0.16)	0.13
Urine										
Osmolality (mOsm/24 h)	500 (182)	599 (167)	108.3 (53.2 to 163.4)	**0.001**	567 (149)	72 (35.2 to 108.7)	**0.001**	521 (198)	−54 (−124 to 14.4)	0.11
Urea (mmol/24 h)	553 (106)	517 (144)	−27 (−84.8 to 29.8)	0.31	460 (90)	−88.5 (−144.8to 32.2)	**0.005**	492 (102)	26.0 (−24.2 to 76.2)	0.28
Free water clearance (mL/min)	−0.87 (0.6)	−1.51 (0.7)	−0.68 (−0.91 to −0.46)	**<0.001**	−1.23 (0.5)	−0.41 (−0.59 to −0.23)	**<0.001**	−0.94 (0.7)	0.38 (0.17–0.58)	**0.002**
Free water reabsorption (mL/min)	0.87 (0.6)	1.51 (0.7)	0.68 (0.46–0.91)	**<0.001**	1.23 (0.5)	0.41 (0.23 to −0.59)	**<0.001**	0.94 (0.7)	−0.38 (−0.58 to −0.17)	**0.002**
	Fractional excretions									
Sodium (%)	0.53 (0.15)	0.52 (0.15)	−0.0 (−0.98 to 0.07)	0.85	0.55 (0.16)	0.01 (−0.07 to 0.1)	0.79	0.48 (0.11)	−0.07 (−0.13 to −0.01)	**0.02**
Potassium (%)	11.7 (3.3)	11.7 (3.5)	0.0 (−1.4 to 1.4)	0.99	12.5 (3.9)	0.76 (−0.9 to 2.5)	0.35	11.2 (3.3)	−1.4 (−2.2 to −0.6)	**0.003**
Urea (%)	24.1 (6.9)	19.9 (5.0)	−3.9 (−6.0 to −1.8)	**0.002**	22.5 (7.6)	−1.5 (−4.8 to 1.7)	0.32	23.6 (7.2)	16.2 (−16.1 to 48.4)	0.29
	RAAS markers									
Plasma										
Renin^c^ (pg/mL)	29 [13–66]	53 [22–128]	182 [120–276]	**<0.01**	47 [25–93]	163 [122–217]	**0.004**	29 [13–62]	−39 [−55 to −17]	**0.004**
Angiotensin II^c^ (pmol/L)	6.9 [3.9–12.5]	19.6 [9.7–39.4]	282 [160–498]	**0.002**	13.0 [8.1–21.0]	188 [118–299]	**0.01**	7.2 [4.6–11.4]	−45 [−64 to −15]	**0.01**
Urine										
Aldosterone (μg/24 h)	14.0 (8.4)	17.2 (6.7)	3.6 (2.2–5.1)	**<0.001**	14.5 (6.9)	−2.4 (3.9)	0.60	14.0 (7.4)	−0.5 (−2.8 to 1.8)	0.64
Angiotensinogen+ (μg/24 h)	6.8 [3.4–14.0]	13.5 [8.3–22.1]	184 [147 to 230]	**<0.001**	12.2 [7.1–20.8]	170 [130–223]	**0.001**	6.3 [2.5 to 16.1]	−48 [−77 to 15.0]	0.10
Renin^c^ (ng/24 h)	8.5 [3.6–20.2]	11.9 [6.0–23.8]	129 [−67 to 251]	0.39	12.5 [6.9–22.7]	137 [−92 to 204]	0.11	14.2 [7.3–27.6]	114 [−69 to 187]	0.58

RAAS, renin-angiotensin-aldosterone system.

All values of baseline urine parameters are the average of urine collection day −3 to day −1; all baseline plasma parameters were collected at day 1.

Bold data indicate significant differences.

a Compared with baseline.

b Compared with end of treatment.

c Geometric mean.

Mean plasma osmolality did not significantly change over time. In comparison, urine osmolality significantly increased from baseline (mean: 500 mOsm/24 h [SD: 182]) at the start of treatment (mean at day 4: 599 mOsm/24 h [SD:167], *P* = 0.001; Table 2) and remained increased at the end of treatment (mean at day 14: 567 mOsm/24 h [SD: 149], *P* = 0.001; Table 2).

Mean plasma urea did not change over time (Table 2). In comparison, urinary 24-hour urea excretion was significantly decreased from baseline (mean: 553 mmol/24 h [SD: 106]) at the end of treatment (mean at day 14: 460 mmol/24 h [SD: 90]; *P* = 0.005; Table 2 and Figure 1). In addition, fractional urea excretion was significantly decreased from baseline (mean 24.1% [SD: 6.9]) at the start of treatment (mean at day 4: 19.9% [SD: 5.0]; *P* = 0.002; Table 2 and Figure 1) but did not significantly change at the end of treatment (mean at day 14: 22.5% [SD: 7.6]; *P* = 0.32; Table 2 and Figure 1) or during follow-up (mean at day 18: 23.6% [SD: 7.2]; *P* = 0.29; Table 2 and Figure 1).

In addition, free water clearance significantly decreased from baseline (mean: −0.87 ml/min [SD: 0.6]) during early dapagliflozin treatment (mean at start of treatment: −1.51 ml/min [SD: 0.7]; *P* = <0.001; Table 2 and Figure 1) and remained decreased at the end of treatment (mean at day 14: −1.23 ml/min [SD: 0.5]; *P* = <0.001; Table 2 and Figure 1). During follow-up, free water clearance significantly increased compared with end of treatment (Table 2 and Figure 1).

### Adaptive Kidney Response: Activation of RAAS Hormones

In general, levels of both plasma and urinary RAAS hormones were acutely increased during treatment with dapagliflozin (Table 2).

Plasma renin level increased significantly from baseline (geometric mean: 29 pg/ml [95% CI: 13–66]) to the start of treatment (geometric mean: 53 pg/ml [95% CI: 22–128]; *P* ≤0.01; Table 2) and remained increased at the end of treatment. During follow-up, geometric mean of plasma renin significantly decreased compared with end of treatment (Table 2).

Mean concentration of plasma angiotensin II was also significantly increased from baseline (geometric mean: 6.9 pmol/l [95% CI: 3.9–12.5]) at day 4 (geometric mean: 19.6 pmol/l [95% CI: 9.7–39.4]; *P* = 0.002; Table 2) and maintained significantly at day 14. Plasma angiotensin II concentration was significantly decreased during follow-up compared with end of treatment (Table 2).

Although urinary renin level was not altered during treatment, 24-hour urinary aldosterone excretion was significantly increased from baseline (mean: 14.0 μg/24 h [SD: 8.4]) at the start of dapagliflozin treatment (mean: 17.2 μg/24 h [SD: 6.7]; *P* <0.001), whereas no differences were observed at the end of treatment or during follow-up (Table 2).

Urinary angiotensinogen level was significantly increased from baseline (geometric mean: 6.8 μg/24 h [95% CI: 3.4–14.0]) at the start of treatment (geometric mean: 13.5 μg/24 h [95% CI: 8.3–22.1]; *P* ≤0.001; Table 2) and remained significantly increased at the end of treatment compared with at baseline. During follow-up, urinary angiotensinogen level returned toward baseline values compared with at the end of treatment (Table 2).

### Effect of Dapagliflozin on UACR and eGFR

As expected, mean eGFR decreased from baseline (mean: 93.1 ml/min per 1.73 m^2^ [SD: 10.3]) during dapagliflozin treatment (mean eGFR at the start of treatment: 89.8 ml/min per 1.73 m^2^ [SD: 12.4]; *P* = 0.06; mean eGFR at the end of treatment: 88.9 [SD: 12.0]; *P* = 0.003) and was reversible after drug discontinuation (Table 3 and Figure 1).

**Table 3:** Changes in renal function (N = 14)

	Baseline	Start of treatment^a^		End of treatment^a^		Follow-up^b^	
	Mean (SD) or geometric mean [95% CI]	Mean (SD) or geometric mean [95% CI]	*P* value	Mean (SD) or geometric mean [95% CI]	*P* value	Mean (SD) or geometric mean [95% CI]	*P* value
UACR (mg/mmol/24 h)	1.35 [0.55–3.32]	1.1 [0.43–2.78]	0.42	1.14 [0.48–2.69]	0.18	1.49 [0.58–3.85]	0.12
Plasma eGFR (CKD-EPI) (mL/min/1.73 m^2^)	93.1 (10.3)	89.8 (12.4)	0.063	88.9 (12.0)	**0.003**	94.7 (10.7)	**0.003**
Percentage change in UACR (%)	—	−19.1 [−52.8 to 38.8]	0.42	−14.7 [−35.4 to 12.5]	0.18	−7.0 [−47.2 to 60.8]	0.12
Change (95% CI) in eGFR (CKD-EPI) (mL/min/1.73 m^2^)	—	−3.3 (−6.8 to 0.2)	0.063	−3.3 (−5.2 to −1.4)	**0.003**	4.9 (2.0–7.8)	**0.003**

CKD-EPI, Chronic Kidney Disease-Epidemiology collaboration; eGFR, estimated glomerular filtration rate; UACR, urinary albumin-to-creatinine ratio.

The values of baseline urine parameters are the average of urine collection day −3 to day −1; all baseline plasma parameters were collected at day 1.

Bold data indicate significant differences.

a Compared with baseline.

b Compared with end of treatment.

Dapagliflozin did not significantly change 24-hour UACR throughout the treatment period (Table 3). Despite no significant changes, the daily percentage change from baseline (geometric mean: 1.35 mg/mmol/24 h [95% CI: 0.55–3.32]) in 24-hour UACR decreased acutely during treatment with dapagliflozin (percentage change at day 4: −19.1% [95% CI: −52.8 to 38.8]; percentage change at day 14: −14.7% [95% CI: −35.4 to 12.5]) and returned toward baseline after treatment discontinuation (percentage change at day follow-up: −7.0% [95% CI: −47.2 to 60.8]; Table 3 and Figure 1).

## Discussion

In this analysis of the DAPASALT study, we assessed kidney responses that control body fluid balance by regulating urinary volumes to counteract the continuous dapagliflozin-induced osmotic diuresis in diuretic-naive people with type 2 diabetes and preserved kidney function.

Previously, we showed that despite increased fractional 24-hour excretion of lithium indicating reduced proximal sodium reabsorption, 24-hour urinary sodium excretion did not change during dapagliflozin treatment.^10^ In the current manuscript, we extend these findings by showing that during standardized sodium intake, multiple intrarenal compensatory mechanisms are activated, including (i) an increase in copeptin, (ii) a decrease in free water clearance that facilitates maintenance of normal urinary volumes; (iii) a decrease in fractional urea excretion; and (iv) activation of RAAS.

The physiological mechanism of renal water conservation is regulated by vasopressin, that is, the antidiuretic hormone (ADH).^11^ ADH stimulates urea and water transport in the inner medullary collecting duct.^16^ Recent data in rodent diabetes models suggest that the osmotic diuresis induced by SGLT2 inhibition is limited by ADH and that this water conservation strategy contributes to successful maintenance of body hydration.^17^ In addition, a recent clinical study that measured levels of copeptin, which is a surrogate of ADH, showed that dapagliflozin not only increased copeptin levels but also activated renal water conservation mechanisms as evidenced by increased urine osmolality and decreased free water clearance.^18^ Therefore, as previously suggested,^11^ the numerical increase in copeptin level in our study, although not significant, is the primary factor driving renal water conservation, which acts as an upstream mechanism of urea-solute and water reabsorption.

Subsequently, urea plays a crucial role in the kidney to concentrate urine, which is fundamental for regulating body fluid status independently of changes in sodium excretion.^19^, ^20^, ^21^ In the inner medulla, urea transporters actively reabsorb urea to create the osmotic gradient required for water reuptake, even during high concentration of tubular sodium and chloride solutes.^11^ Hence, urea-driven urine concentration is an important component to limit osmotic diuresis and prevent dehydration. Various reports suggest that SGLT2 inhibition similarly induces renal urea reabsorption to limit sodium and glucose-driven osmotic diuresis.^11^ We found a significant decrease in fractional urea excretion, which supports this urea-driven water conservation concept, also known as the “aestivation” theory.^11^ In this light, hepatic urea production should be increased^22^^,^^23^; however, hepatic urea production could not be measured in this study.

Our study expands these findings by showing that the increase in urine osmolality and decrease in free water clearance occur rapidly, are maintained during treatment, and are reduced after washout during standardized sodium diet. Together with the decrease in fractional 24-hour urea excretion, we hypothesize that SGLT2 inhibition triggers ADH-mediated renal water conservation by increasing urea-solute and water reabsorption during the urine-concentrating process in the kidney.

Finally, regarding RAAS, nearly all studies with SGLT2 inhibitors have shown increments in systemic RAAS hormones during SGLT2 treatment.^24^^,^^25^ We extend these previous findings by showing that this response is quickly initiated, maintained during treatment, and reduced after washout during standardized sodium diet. Both systemic and intrarenal parameters of RAAS were acutely increased, which could to a certain extent be a response to the reduction in BP.^10^ At follow-up, however, all RAAS parameters had returned to baseline values despite a persistent reduction in BP.^10^ Thus, changes in RAAS parameters may also reflect intrarenal RAAS effects in addition to the effects of systemic hemodynamics. As such, urinary angiotensinogen can serve as an index of intrarenal RAAS activity and is determined by the filtration of plasma angiotensinogen through the damaged glomeruli and production of local angiotensinogen in the proximal tubules in some animal models.^26^^,^^27^ Urinary angiotensinogen is associated with glomerular dysfunction and positively correlated with albuminuria.^28^ In the current study, dapagliflozin significantly increased urinary angiotensinogen whereas UACR was rather tended to be decreased and eGFR decreased. Therefore, these data indicate that urinary angiotensinogen is not associated with its glomerular filtration, at least not in these patients. Indeed, previous studies have shown that intrarenal generation of angiotensinogen has also significant impact on urinary angiotensinogen levels in some pathophysiological conditions.^29^^,^^30^

The RAAS also acts to limit excessive fluid loss by increasing sodium reabsorption in the distal segments of the kidneys, possibly reflected in our study by an increase in urinary aldosterone excretion, despite all people using angiotensin receptor blockers. Despite changes in plasma and urinary aldosterone concentrations, we did not observe changes in serum potassium concentrations (results not reported) and urinary potassium excretion (Table 2 and Supplementary Table S1).

In cardiovascular outcome trials, SGLT2 inhibitors reduce renal and cardiovascular risks regardless of background RAAS inhibitor use without causing additional side effects, emphasizing that clinical benefits of SGLT2 inhibitors are independent of baseline RAAS activation in humans.^2^, ^3^, ^4^, ^5^ Rather, RAAS activation during angiotensin-converting enzyme inhibition or angiotensin receptor blockade may favor the renoprotective effects of the nonclassic RAAS pathways, including angiotensin 1-7 production and the activation of type-2-angiotensin-II receptor and Mas receptors.^31^

Regarding their mechanism of action, SGLT2 inhibitors differ from traditional diuretics, such as loop or thiazide diuretics, as published before.^32^^,^^33^ This study provides additional data that differentiate SGLT2 inhibitors from traditional diuretics. Loop diuretics decrease body fluid volume and increase acute renal dysfunction in a dose-dependent manner.^34^ In contrast, our study shows that SGLT2 inhibitors induce a range of compensatory mechanisms to prevent dehydration and maintain adequate body fluid volume, which may help to attenuate the risk of acute kidney injury.^35^

Taken together, these 4 response mechanisms play an important role to maintain body fluid balance and safe use of SGLT2 inhibitors. In addition, SGLT2 inhibitors have shown renoprotective effects in people with and without diabetes in the 2 large dedicated chronic kidney disease outcome trials CREDENCE and DAPA-CKD.^6^^,^^7^ The responsible mechanisms are not yet fully understood, but they have at least in part been attributed to kidney hemodynamic changes, such as decreases in intraglomerular pressure and hyperfiltration.^8^^,^^9^^,^^36^ Clinically, this is manifested by SGLT2 inhibitor-induced reductions in eGFR and albuminuria after 4 to 8 weeks of treatment.^36^, ^37^, ^38^ So far, this is the first mechanistic clinical trial that assessed the time course of the effect on eGFR and UACR during treatment with dapagliflozin. Our acutely observed reductions in both suggest that the effect on both eGFR and albuminuria in long-term trials emerges directly after treatment initiation with an SGLT2 inhibitor. The lack of a significant effect on UACR may be explained by the low baseline UACR coupled with a large day-to-day interindividual variation.^38^ We also show that the effects of dapagliflozin on eGFR and UACR dissipate after a few days of washout, confirming a renal hemodynamic effect, particularly as systemic BP was still decreased, implicating a renal-specific effect.

Strengths of our study include standardization of sodium intake and detailed measurements of kidney responses with repeated 24-hour urinary collections. We acknowledge some limitations. We performed this study in a homogeneous group of people with type 2 diabetes and preserved kidney function who were all using an angiotensin receptor blocker thereby avoiding disease heterogeneity and potential confounding by preexisting kidney disease. The carefully determined group may limit the generalizability of our findings. Also, the size of our study cohort was small, which limits the precision of the effect estimates for the secondary and exploratory end points and resulted in nonsignificant findings despite large percentage changes. Finally, the open-label design does not allow definitive conclusions. Our results should therefore be considered hypothesis generating.

In conclusion, we demonstrated that dapagliflozin-induced osmotic diuresis triggers different kidney adaptive mechanisms to maintain volume and sodium balance in people with type 2 diabetes and preserved kidney function.

## Disclosure

This study was funded by AstraZeneca. MHAM has acted as a speaker/consultant for AstraZeneca, Eli Lilly, Novo Nordisk, and Sanofi; all honoraria are paid to his employer (AUMC, location VUMC). PJG, CK, and AH are employees and shareholders at AstraZeneca. DHvR has acted as a consultant and received honoraria from Boehringer Ingelheim, Eli Lilly, Merck, Novo Nordisk, Sanofi, and AstraZeneca and has received research operating funds from Boehringer Ingelheim, Eli Lilly Diabetes Alliance, AstraZeneca, and Novo Nordisk; all honoraria are paid to his employer (AUMC, location VUMC). HJLH is a consultant for AbbVie, AstraZeneca, Bayer, Boehringer Ingelheim, Chinook, CSL Pharma, Gilead Sciences, Janssen, Merck, Mundipharma, Mitsubishi Tanabe, Novo Nordisk, and Retrophin and reports receiving research support from AbbVie, AstraZeneca, Boehringer Ingelheim, and Janssen. All the other authors declared no competing interests.

## Supplementary Material

Supplementary file (PDF)

**Table S1.** Changes in primary and secondary outcomes (*N* = 14).

**Figure S1.** Study design.
